# A bibliometric analysis of autism spectrum disorder signaling pathways research in the past decade

**DOI:** 10.3389/fpsyt.2024.1304916

**Published:** 2024-02-12

**Authors:** Kaifeng Lyu, Jiangshan Li, Min Chen, Wu Li, Wei Zhang, Meichao Hu, Yuxing Zhang, Xiang Feng

**Affiliations:** ^1^ Faculty of Chinese Medicine, Macau University of Science and Technology, Macau, China; ^2^ College of Acupuncture, Moxibustion, Massage, and Rehabilitation, Hunan University of Traditional Chinese Medicine, Changsha, China; ^3^ Faculty of Chinese Medicine and State Key Laboratory of Quality Research in Chinese Medicines, Macau University of Science and Technology, Macau, China; ^4^ Pediatrics One, The First Affiliated Hospital of Heilongjiang University of Traditional Chinese Medicine, Harbin, China; ^5^ Rehabilitation Department, Chifeng Obstetrics and Gynecology Hospitalal, Chifeng, China

**Keywords:** signaling pathways, ASD, bibliometric analysis, Citespace, VOSviewer

## Abstract

**Background:**

This study employs bibliometric methods to comprehensively understand the fundamental structure of research about Autism Spectrum Disorder (ASD) Signaling Pathways by examining key indicators such as nations, institutions, journals, authors, and keywords.

**Methodology:**

We utilized the WoScc database to retrieve literature relevant to ASD Signaling Pathways published between 2013 and 2023. Through visual analysis and tools like CiteSpace and VosViewer, we explored nations, institutions, journals, authors, and keywords, thereby constructing relevant networks.

**Results:**

26 The study encompasses 1,396 articles, revealing a consistent increase in publications. The United States, China, and Germany are leading nations in this literature. Regarding research institutions, the University of California system and Eric Klann have garnered significant attention due to their substantial contributions to the field of ASD Signaling Pathways. Most relevant research is published in the journal “Molecular Autism.” Research interests are concentrated across various themes, including “elevating neuronal β-catenin levels,” “Tunisian children,” “*Fmr1* knockout (KO) mice,” “*de novo* mutations,” “autistic children,” “local translation,” “propionic acid-induced mouse models,” “neurosystems,” “glucose metabolism,” and “neuronal migration.” Future research may emphasize exploring aspects such as gut microbiota, genes, stress, maternal immune activation, memory, and neurodevelopmental disorders of ASD.

**Conclusion:**

This study, through bibliometric analysis of key indicators such as nations, institutions, journals, authors, and keywords, provides a comprehensive overview of the current state of research on ASD Signaling Pathways. These investigations predominantly focus on molecular mechanisms, animal model studies, population-based research, and the structure and function of neurosystems. Future research directions are also clearly proposed. First, in-depth research on the genes and neurodevelopmental disorders associated with ASD will continue to reveal the genetic basis and provide support for precise treatments. At the same time, attention to the gut microbiota will help explore its association with ASD, which may provide clues for new treatments. In addition, the relationship between stress and ASD will become the focus of research to understand better the emotional and behavioral characteristics of ASD patients in stressful situations. Maternal immune activation will also be further studied to explore how environmental factors influence the risk and development of ASD. Finally, a deeper understanding of the cognitive functions of patients with ASD, especially memory and learning, will help develop individualized treatment strategies to improve patients’ quality of life. These directions will work together and are expected to provide a more comprehensive understanding of Signaling Pathways research in ASD and provide new ideas and opportunities for future intervention and treatment.

## 1 Introduction

Autism Spectrum Disorder (ASD) represents a constellation of intricate neurodevelopmental disorders. Typically manifesting in early childhood, its cardinal clinical manifestations encompass impaired communication abilities, deficient social interaction, and stereotyped and repetitive behaviors. The Diagnostic and Statistical Manual of Mental Disorders, Fifth Edition (DSM-5) (1) classifies ASD, Asperger’s syndrome, childhood disintegrative disorder, and unspecified pervasive developmental disorders all under the rubric of ASD. As per the World Health Organization’s (WHO) (2) estimations, it is projected that by 2022, approximately one in every 100 children globally will have ASD. Currently, the global prevalence of this disorder is on a relentless ascent. In 2020, the Centers for Disease Control and Prevention (CDC) released the latest epidemiological study, encompassing data from 11 states, revealing that 1 in 44 children under the age of eight has been diagnosed with ASD, marking an increase of 30% from estimates in 2010 (3). Research reports from Asia (4), Europe (5), and Africa (6) indicate an average prevalence rate among ASD patients ranging from 0.48% to 3.13%. Children with ASD impose long-term medical and educational costs on society and families. In the United States, caring for a child with ASD entails an annual expenditure exceeding $20,000 for medical and educational purposes (7). ASD has thus become a long-standing concern for clinical professionals.

The etiology of ASD involves various factors, with particular attention drawn to research on ASD signaling pathways. Firstly, these pathways might play a pivotal role in the early diagnosis of ASD. Understanding the abnormalities or characteristic changes in these pathways makes it possible to establish more accurate diagnostic markers, providing a window for early intervention. Secondly, research on ASD signaling pathways could potentially drive the development of therapeutic strategies. Understanding these pathways may offer new therapeutic targets or intervention methods for precision medicine. Finally, delving deeper into these signaling pathways may contribute to expanding our understanding of the etiology and theoretical cognition of ASD, better distinguishing between different subtypes, identifying potential shared mechanisms, and paving the way for novel directions in future research. Over the past decade, research about the genetic correlates of ASD has witnessed remarkable advancements, progressing from monoclonal gene studies to contemporary large-scale investigations employing whole-genome sequencing (WGS) (8). Numerous highly reliable and reproducible risk genes have been identified, including *Shank3* (9), *Cntnap2* (10), and *Nlgn3* (11), among others. These genes are associated with neuronal connectivity, synaptic function, and neurodevelopment. Studies suggest that these anomalies may lead to disruptions in information transmission, subsequently affecting social skills, learning, and memory (12). Concurrently, researchers are exploring multiple neurodevelopmental Signaling Pathways, such as *Wnt* (13), *Sonic Hedgehog* (14), *mTOR*, and others (15), to elucidate their roles in the pathogenesis of ASD, which may result in abnormal brain structure and function. Additionally, some scholars posit that immune system aberrations and inflammatory responses may be implicated in the pathogenesis of ASD, with significant alterations noted in the expression levels of cytokines (16) and apoptosis regulatory factors (17), among others, within ASD patients. Research indicates an overactivation of immune pathways (18) in ASD patients, encompassing abnormalities in immune cells such as T cells, B cells, and macrophages (19). These aberrant immune responses may culminate in neuroinflammation, neuronal damage, and abnormal brain structure and function development. Beyond genetic, neurobiological, and immune factors, environmental factors are also considered potential influencers in the pathogenesis of ASD. Some studies have found associations between prenatal exposure to antidepressants (20), antibiotics (21), or other medications (22) and an increased risk of ASD. Furthermore, certain chemical substances (23), such as mercury and organic chlorides, have also been found to be associated with ASD.

We employ CiteSpace (24) and VosViewer (25) to delineate this research domain. Citespace, a bibliometric analysis tool, is not open source. It was developed by Dr. Chaomei Chen’s team at Tsinghua University and is provided to the academic community free of charge. However, its source code has not been publicly disclosed. On the other hand, VosViewer, which is used for visual analysis, is an open-source software developed by the Centre for Science and Technology Studies (CWTS) at Leiden University. The source code for VosViewer is openly stored in its GitHub repository. The source code is available at https://github.com/neesjanvaneck/VOSviewer-Online.git.

As the global prevalence of ASD steadily rises, these distinct indicators play a pivotal role in evaluating research dynamics and worldwide impact. They aid in discerning variations in ASD incidence rates, identifying environmental influences in particular geographic regions, setting research priorities, and fostering collaboration opportunities among diverse research institutions. It encompasses but is not limited to (1) comprehensive statistical analysis, including annual publication counts, growth patterns, countries of origin, institutions, authors, and journals. (2) Citation analysis, including common citations among journals; (3) Co-occurrence studies, including visual representations of frequently used keywords; (4) Temporal evaluation of emerging keywords over time. The search time was from January 2013 to October 1, 2023. The literature search covered a variety of sources, including but not limited to journal articles, theses, and conference proceedings included in the database. The search language is limited to English, and there are no particular restrictions on other search conditions. English, as the primary language for international academic communication, enhances the reliability and consistency of data when utilized in scholarly literature. This aids in mitigating potential ambiguities or misunderstandings that may arise due to language differences. However, it may also result in the inadvertent neglect of non-English literature.

## 2 Materials and methods

### 2.1 Data sources and retrieval strategies

Due to the authoritative nature of database citations, our study predominantly utilized the WOSCC database as the primary data source. Firstly, WOScc offers comprehensive disciplinary coverage, encompassing natural sciences, social sciences, and humanities, enabling Citespace to conduct interdisciplinary literature analysis and provide a more comprehensive research perspective. Secondly, WOScc possesses detailed literature metadata and citation data, including rich bibliographic information such as authors, abstracts, keywords, author affiliations, journal sources, and citation relationships among documents. This is crucial for Citespace’s citation network analysis and scientometric research. Additionally, WOScc maintains high-quality and standardized data, ensuring the credibility of the literature and consistency of information. Its provision of standardized data formats and structures aids Citespace in more effectively processing and analyzing literature information. Lastly, WOScc has earned the trust and widespread adoption by researchers, journals, and institutions as a widely recognized and extensively used database within the academic community. This positions it as an ideal data source for Citespace’s literature metric analysis and visualization.

We employed precise title searches, using terms derived from the ClinicalTrials.gov database and the National Library of Medicine (NLM) database as the basis, to ensure that our publication retrieval task yielded comprehensive and reliable results. Our primary search string comprised “TS=(Signaling Pathways) AND TS= [(autism) OR (autism spectrum disorder) OR (ASD)].”

### 2.2 Research methods

**Figure 1:**
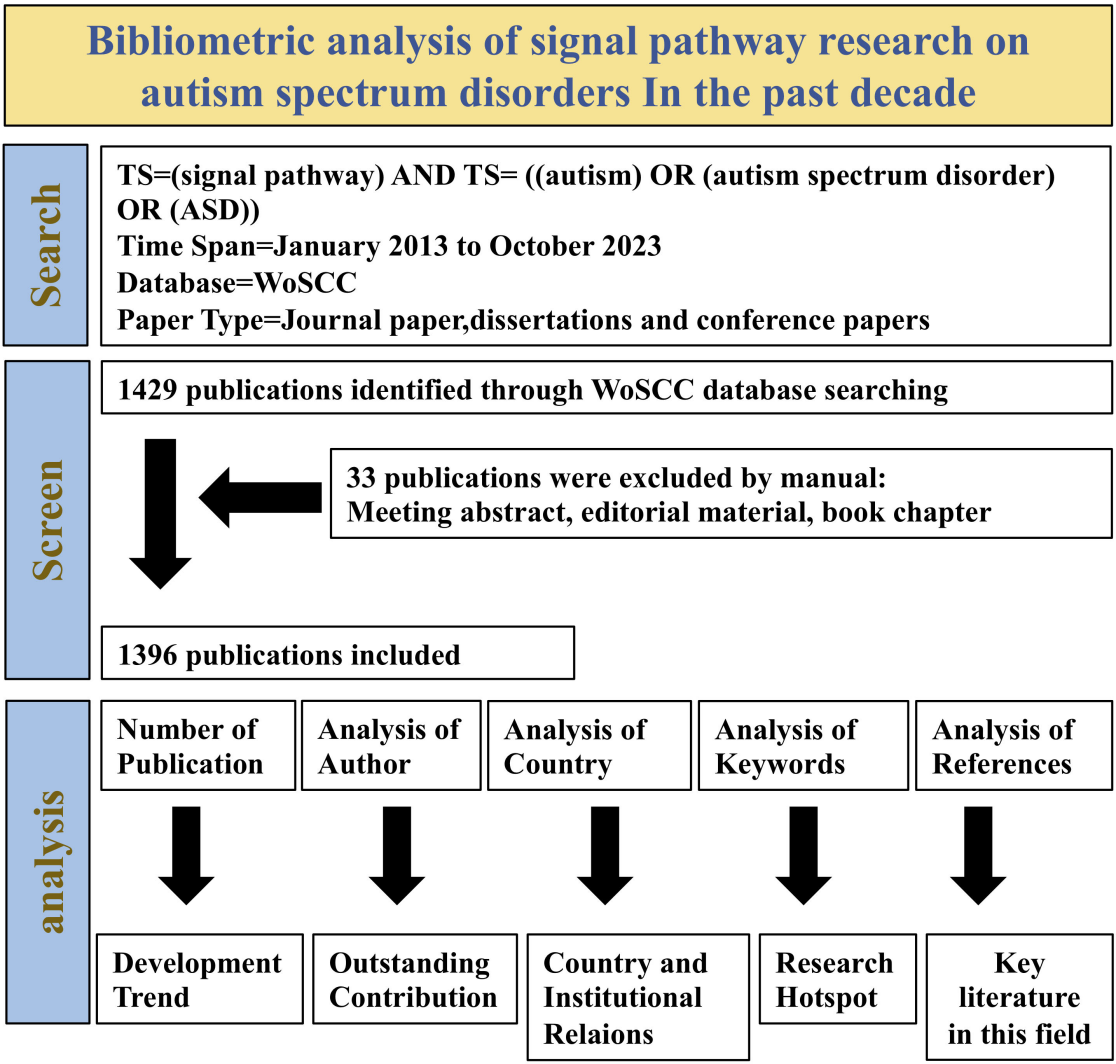
Prisma flow diagram.

In order to conduct an in-depth bibliometric analysis, we exported citation data and complete records in “plain text format” and saved them with the naming convention “download_XXX.” Subsequently, we extracted text-format data from WoSCC, including titles, authors, abstracts, keywords, and the source of the literature. This data was then imported into VOSviewer and CiteSpace software for bibliometric and visualization analysis (**Figure 1**).

Firstly, within CiteSpace, we harnessed its robust literature visualization capabilities to construct a knowledge network graph. Each node in the graph represents distinct countries/regions, institutions, or authors. Typically, the size of nodes reflects their frequency of appearance or citation, while different colors signify different entities. Connections between nodes indicate collaboration, co-occurrence, or citation relationships. This approach unveiled the primary research countries, institutions, topics, hotspots, and developmental trends within the research domain. By configuring parameters such as node size, color, and links, we were able to elucidate the relationships and significance among the literature. Betweenness centrality refers to the number of times a node acts as the shortest bridge between other nodes, serving as one of the metrics to measure the importance of nodes within a network. It gauges the extent to which papers, authors, or keywords act as bridging components among diverse fields, topics, or scholars within the academic network. Nodes with high betweenness centrality imply pivotal intermediary roles in disseminating academic information and facilitating connections between different themes or domains, akin to critical hubs in information dissemination.

Subsequently, in VosViewer, we employed its bibliometric analysis capabilities to visualize journal trends and author collaborations within the literature dataset. This methodology aids in identifying influential journals within the field and tracking changes in their prominence over time. Additionally, it facilitates a more profound exploration of the most tightly-knit collaborating author groups in the domain, unveiling the structure of academic collaboration networks and key contributors.

## 3 Results

### 3.1 Bibliometric analysis by publication year

**Figure 2:**
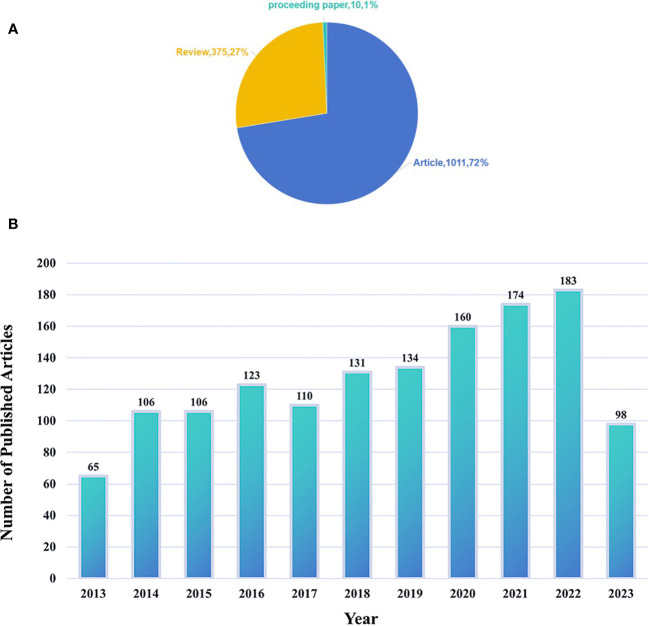
**(A)** Type of publications. **(B)** The combination chart of the number of annual.

The distribution of relevant literature over time is analyzed yearly as a time interval for statistical purposes. From January 2013 to October 1, 2023, the WOSCC database publicly indexed 1,396 articles in this field. These articles involve 67 countries/regions, 222 institutions, 61 journals, and 449 authors. The types of publications included are as follows: articles (1,011), review articles (375), and conference papers (10). Articles and review articles collectively account for 99% of the publications (**Figure 2A**). The number of publications generally increased over time, with an annual growth trend that fits the curve y = 11.285x – 22638 R² = 0.915 (**Figure 2B**). The equation represents a linear growth model. The high R² value indicates a robust fit of the model to the data. A value close to 1 suggests that the fitted curve effectively explains a significant portion of the data variance. Hence, this growth curve can be considered to reasonably meet the standard criteria for fitting. As of October 1, 2023, 98 articles were published for the entire year.

The **Table 1** lists the top five countries for publishing relevant research. These five countries have published 869 articles (62.2% of the total). The United States has published the most pertinent research (466 articles, accounting for 33.3%), and it also has the highest centrality (centrality = 0.5), indicating extensive international collaboration involving U.S. institutions.

**Table 1:** Top five countries in terms of publications and centrality.

Ranking	Country/region	Documents	Centrality
1	United States	466	0.5
2	China	191	0.03
3	Germany	76	0.09
4	Canada	69	0.11
5	England	67	0.08

### 3.2 Metrology analysis of institutions and country

**Figure 3:**
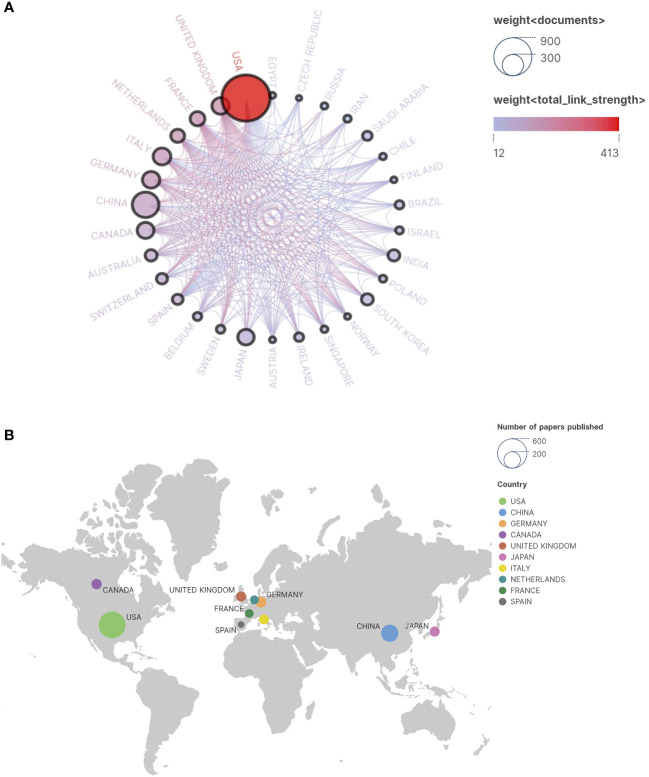
**(A)** Cooperation Network Country/Region. In the illustration, individual circles depict countries contributing to published research, while interconnecting lines represent collaborative efforts among these nations. The size of each circle corresponds to the volume of relevant research publications originating from that country. The closer the circle is to the red hue, the greater the number of articles that country has published in collaboration with others. Thicker lines denote stronger collaboration and closer partnerships between countries. **(B)** Publication country and regional distribution map.

**Figure 4:**
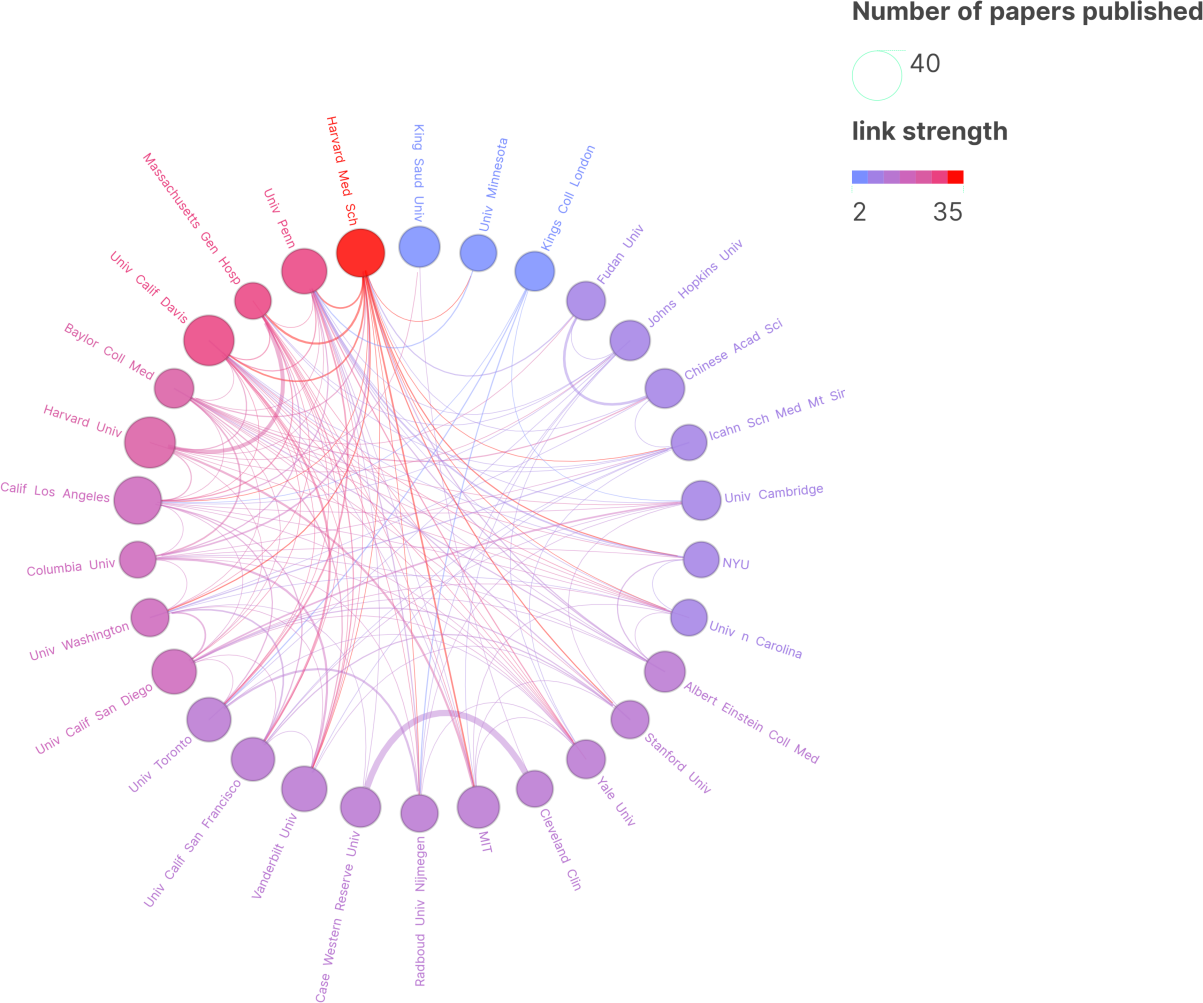
Research Institution Map. In the graphical representation, each circle signifies the quantity of research publications attributed to a particular institution, with connecting lines illustrating collaborative endeavors between these institutions. The proximity of the circle to the shade of purple correlates with the institution’s volume of collaborative research articles. The thickness of the lines denotes a higher degree of collaboration and closer partnerships between institutions.

The research covers a global scope involving collaboration from 67 countries. The **Figure 3A** illustrates the collaborative relationships among these countries. The world collaboration map (**Figure 3B**) demonstrates the interactions among various nations, highlighting active collaborations between the United States and China, Germany, and the United Kingdom. The **Figure 4** portrays the collaboration involving approximately 222 research institutions. While China ranks second in the total number of papers, indicating considerable activity in the relevant field, the top ten institutions in the **Table 2** may not comprehensively represent China’s overall contributions in this domain. Numerous research institutions in China collectively make significant contributions, accounting for approximately 6% of 222 research institutions. This broader participation strategy implies that individual institutions may not surpass those of other countries in terms of publication quantity in specific fields, leading to an observed dilution effect. Therefore, a comprehensive assessment of China’s position and impact on research necessitates considering contributions at the national level and collaboration among multiple institutions.

**Table 2:** Top Ten institutions in terms of publications and citations.

Ranking	Institution	Documents	Centrality
1	University of California System	94	0.19
2	Harvard University	56	0.19
3	UDICE-French Research Universities	40	0.11
4	Harvard Medical School	30	0.02
5	Institut National de la Sante et de la Recherche Medicale (Inserm)	29	0.03
6	Centre National de la Recherche Scientifique (CNRS)	27	0.07
7	Massachusetts Institute of Technology (MIT)	24	0.03
8	University of California Davis	24	0.03
9	National Institutes of Health (NIH) - USA	24	0.12
10	University of Texas System	20	0.05

### 3.3 Journal analysis

**Figure 5:**
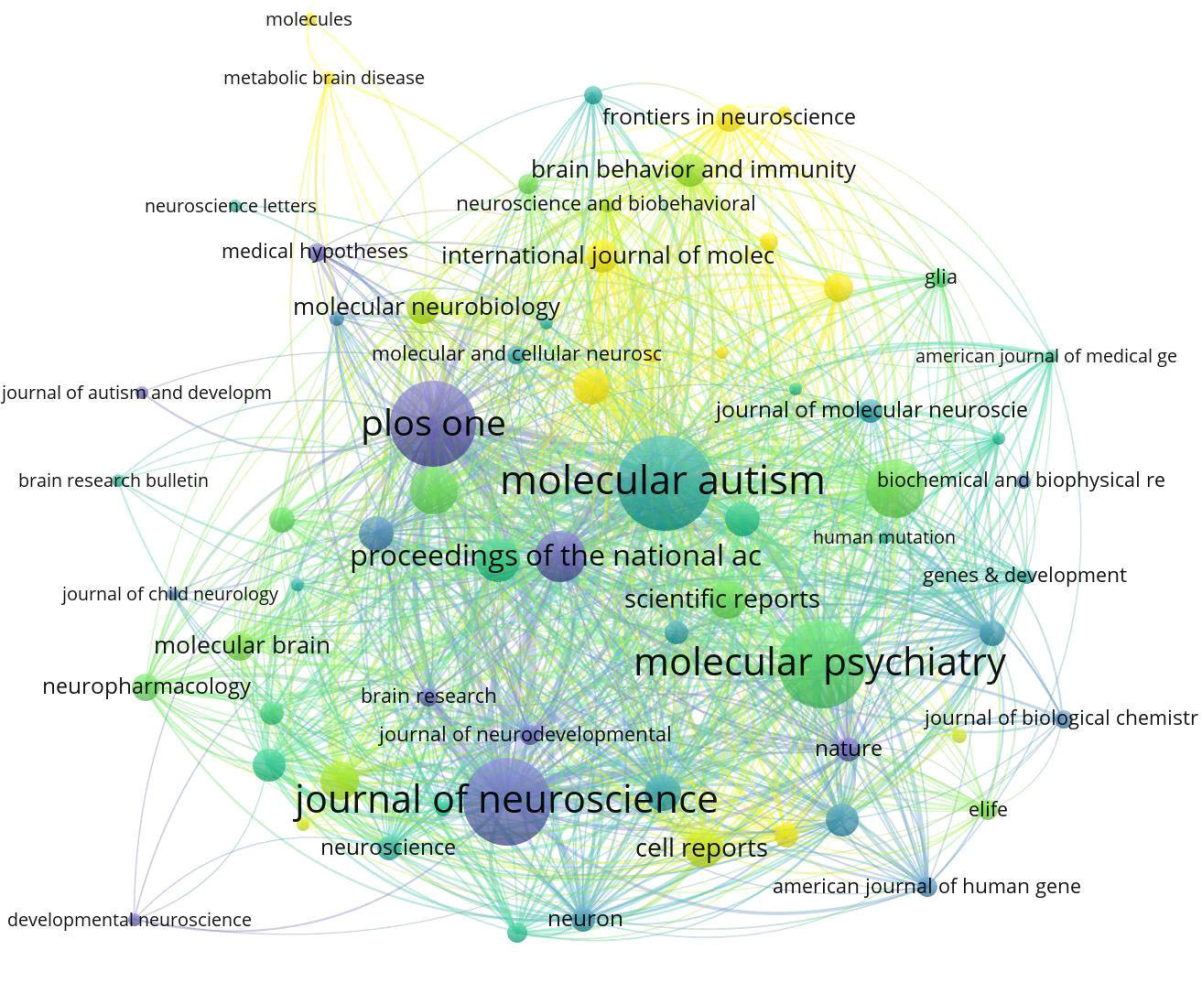
Average number of years that research on ASD-related Signaling Pathways has been published in high-volume journals. The nodes depicted in The **Image 5** symbolize diverse journals. The magnitude of each node corresponds to the volume of associated papers it has published, while the color-coded legend signifies the average publication year of all articles within the journals. Journals highlighted with a yellow background denote recent engagement in research within a specific field. Conversely, those journals with a purple background may suggest a decrease in interest regarding the topic during the analyzed time frame.

The **Figure 5** depicts the temporal assessment of journal output. It is worth noting that comprehensive journals such as Plos One and the Journal of Neuroscience accepted this topic earlier, with the average publication year between 2013 and 2016. In contrast, some professional journals related to ASD spectrum disorders and signaling pathways, such as Frontiers Genetics and Frontiers in Neuroscience, have recently begun to focus on research in this area.

The **Table 3** presents a compilation of the top ten high-output journals. Among them, “Molecular Autism” has the highest number of publications and citations, with 48 articles and 2,144 citations. It is followed by the journal “Molecular Psychiatry,” which has 46 articles and 3,138 citations.

**Table 3:** Top ten journals in terms of publications.

Ranking	Journal	Documents	Citations	Citations/paper
1	Molecular Autism	48	2144	44.6667
2	Molecular Psychiatry	46	3138	68.2174
3	Journal of Neuroscience	41	2650	64.6341
4	Plos One	40	1821	45.525
5	International Journal of Molecular Sciences	39	939	24.0769
6	Frontiers in Cellular Neuroscience	36	1131	31.4167
7	Translational Psychiatry	27	553	20.4815
8	Frontiers in Molecular Neuroscience	26	681	26.1923
9	Frontiers in Psychiatry	22	263	11.9545
10	Proceedings of the National Academy of Sciences of the United States of America	22	2013	91.5

### 3.4 Biometric analysis of author cooperation

**Figure 6:**
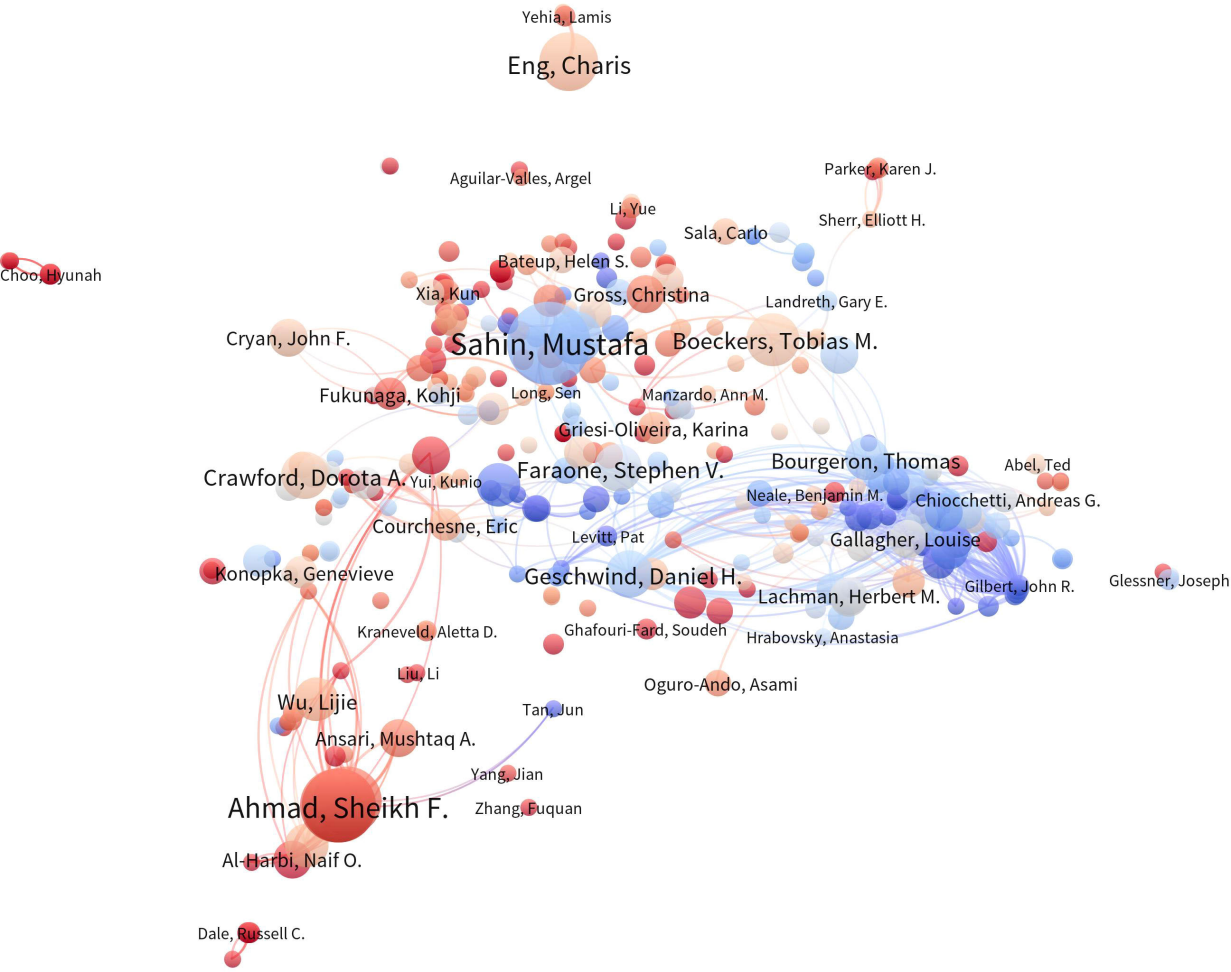
Author Co-occurrence Map. In the author co-occurrence analysis map, the size of each node signifies the quantity of publications authored by the respective author, while the innermost color represents the average publication year of the author’s relevant documents. The links between nodes denote the presence of collaborative relationships among authors, with the thickness of the connections indicating the strength of cooperation among them.

The **Figure 6** displays a co-authorship network analysis of significant authors in the literature. The **Table 4** provides a summary of the top 10 most productive authors. Notably, 60% of these authors are from the United States. With ten papers and 1,153 citations, Eric Klann emerges as the most prolific author, followed by Christina Gross (7, 95) and Claudia Bagni (6, 556), underscoring their significance in advancing research in this field. However, it is worth noting that the number of papers published by scholars in this field is generally limited (**Table 4**).

**Table 4:** Top ten authors.

Ranking	Author	Documents	Citations	Citations/paper	Country
1	Eric Klann	10	1153	115.3	United States
2	Christina Gross	7	95	13.5714	United States
3	Claudia Bagni	6	556	92.6667	Switzerland
4	Gary J. Bassell	6	264	44	United States
5	Yuri Bozzi	6	148	24.6667	Italy
6	Margaret A. Pericak-Vance	6	724	120.6667	United States
7	Maria Vincenza Catania	5	174	34.8	Italy
8	Michael L. Cuccaro	5	723	144.6	United States
9	Dorota A. Crawford	5	218	43.6	Italy
10	Stephen V. Faraone	5	206	41.2	United States

### 3.5 Analysis of co-cited references

**Figure 7:**
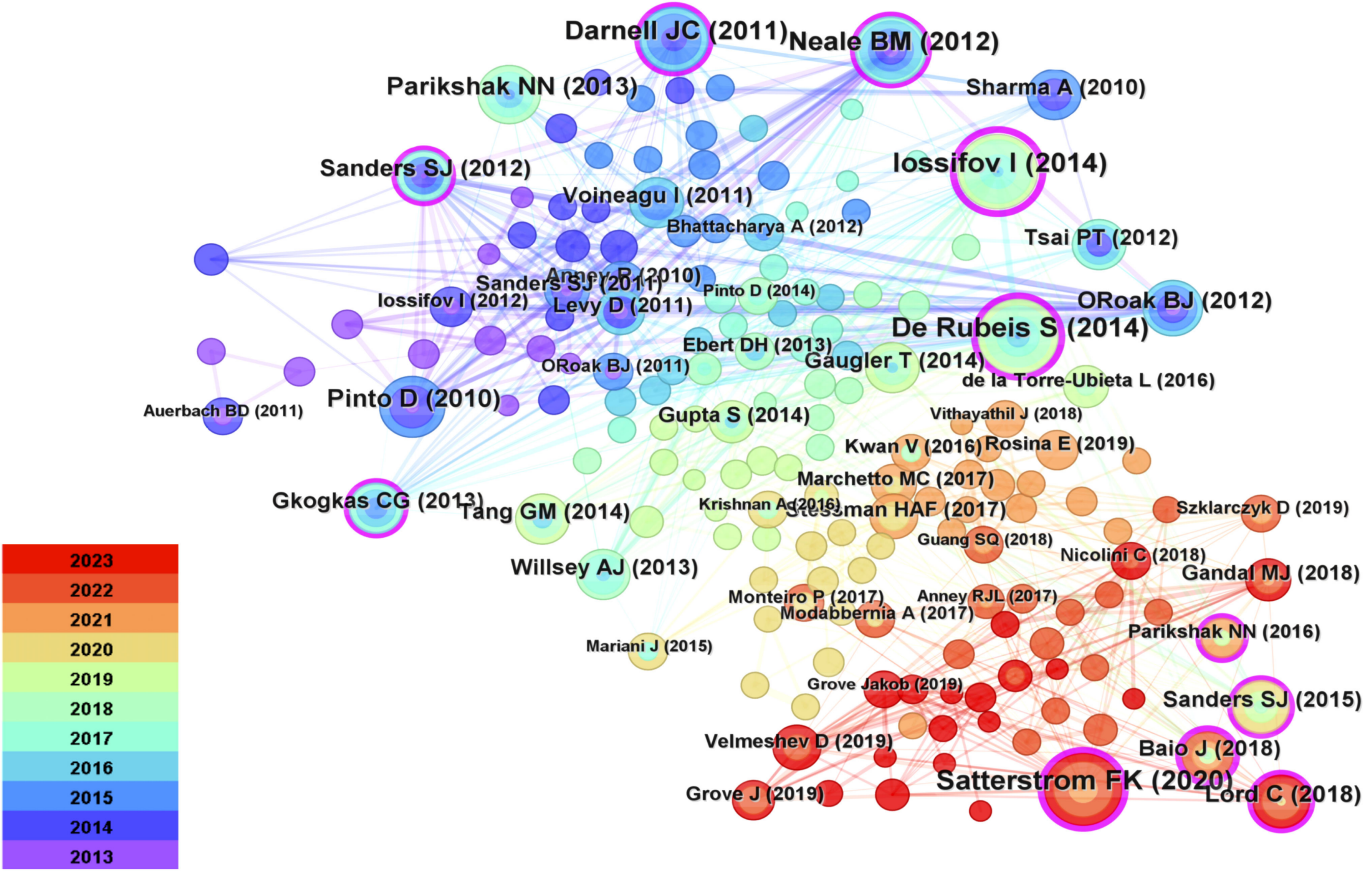
Reference Diagrams for Common References. Cited documents serve as a reflection of the comprehensive knowledge foundation within the field, with highly cited documents often regarded as seminal classics. Each node within the network denotes a citation, where the node’s size corresponds to its frequency of co-citations. Interconnecting lines between nodes signify instances of co-citation within the literature.

Using CiteSpace 6.2.R4, a co-citation reference map was constructed (**Figure 7**), which includes 194 nodes and 795 links. From a broader perspective, the nodes exhibit dispersion, indicating that only a few authors maintain close collaborations. This underscores the need to strengthen scientific partnerships among scholars to foster more profound developments in this field.

The **Table 5** presents summaries of the top five most frequently cited references. Citations refer to the number of times an author’s published papers are referenced by other scholarly articles. Citespace utilizes citation data from the literature database to compute the total count of times specific authors’ papers have been cited by other works. This method aids in gauging an author’s influence and the extent of their research impact. Documents represent the quantity of papers authored by an individual. Citespace, relying on the authorship information within the literature database, tallies the number of papers attributed to specific authors. This metric serves as a gauge for an author’s activity and contributions within the field. Among them, the three most frequently cited references are Iossifov I (2014) (26) (cited 38 times), De Rubeis S (2014) (27) (cited 36 times), and Satterstrom FK (2020) (28) (cited 35 times).

**Table 5:** Top five most co-cited references.

Rank	Co-Cited References	Citations
1	Iossifov I, 2014, NATURE, V515, P216, DOI 10.1038/nature13908	38
2	De Rubeis S, 2014, NATURE, V515, P209, DOI 10.1038/nature13772	36
3	Satterstrom FK, 2020, CELL, V180, P568, DOI 10.1016/j.cell.2019.12.036	35
4	Neale BM, 2012, NATURE, V485, P242, DOI 10.1038/nature11011	30
5	Darnell JC, 2011, CELL, V146, P247, DOI 10.1016/j.cell.2011.06.013	28

### 3.6 Keyword analysis

**Figure 8:**
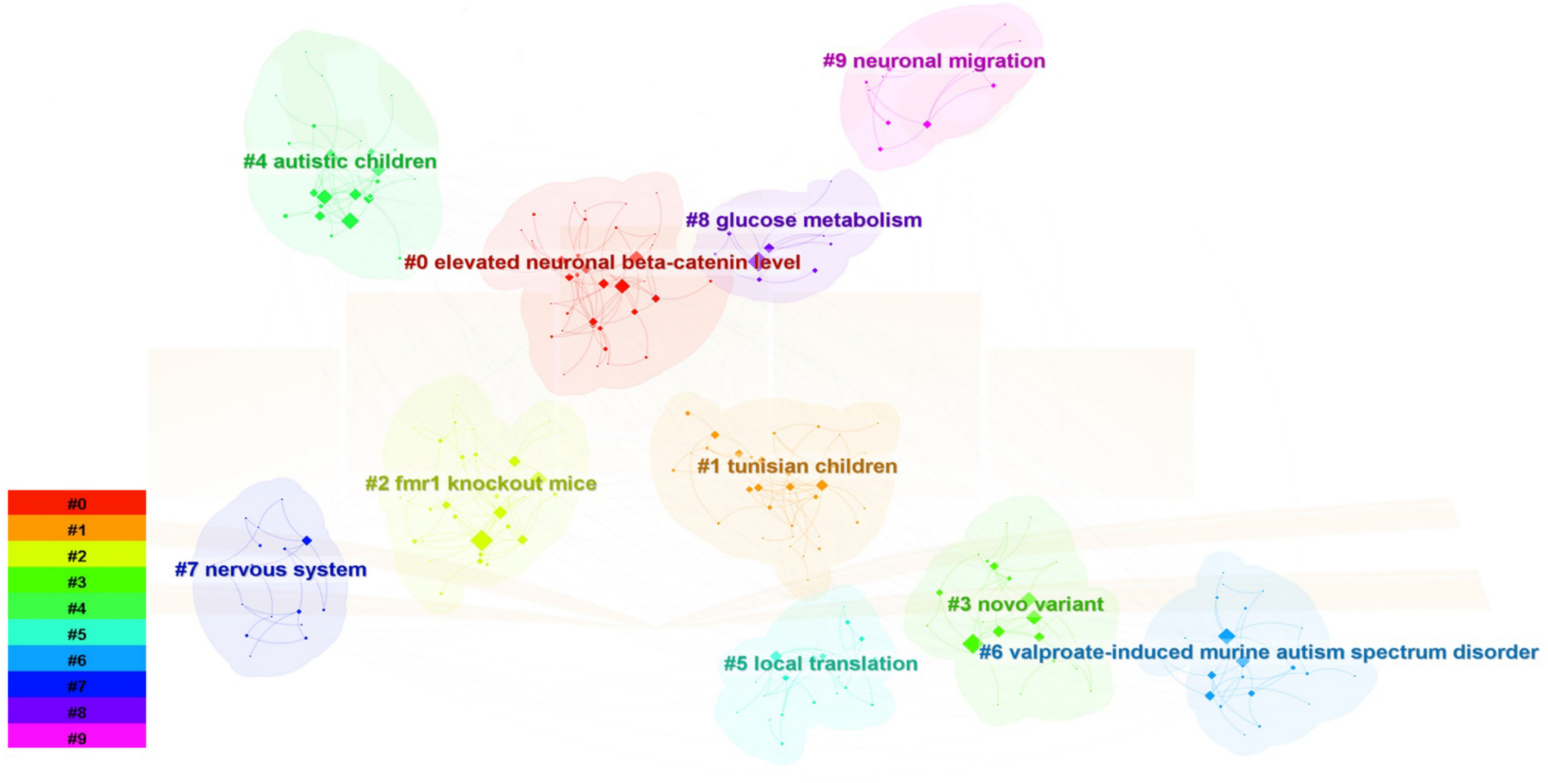
Keyword Co-occurrence Diagram.

**Figure 9:**
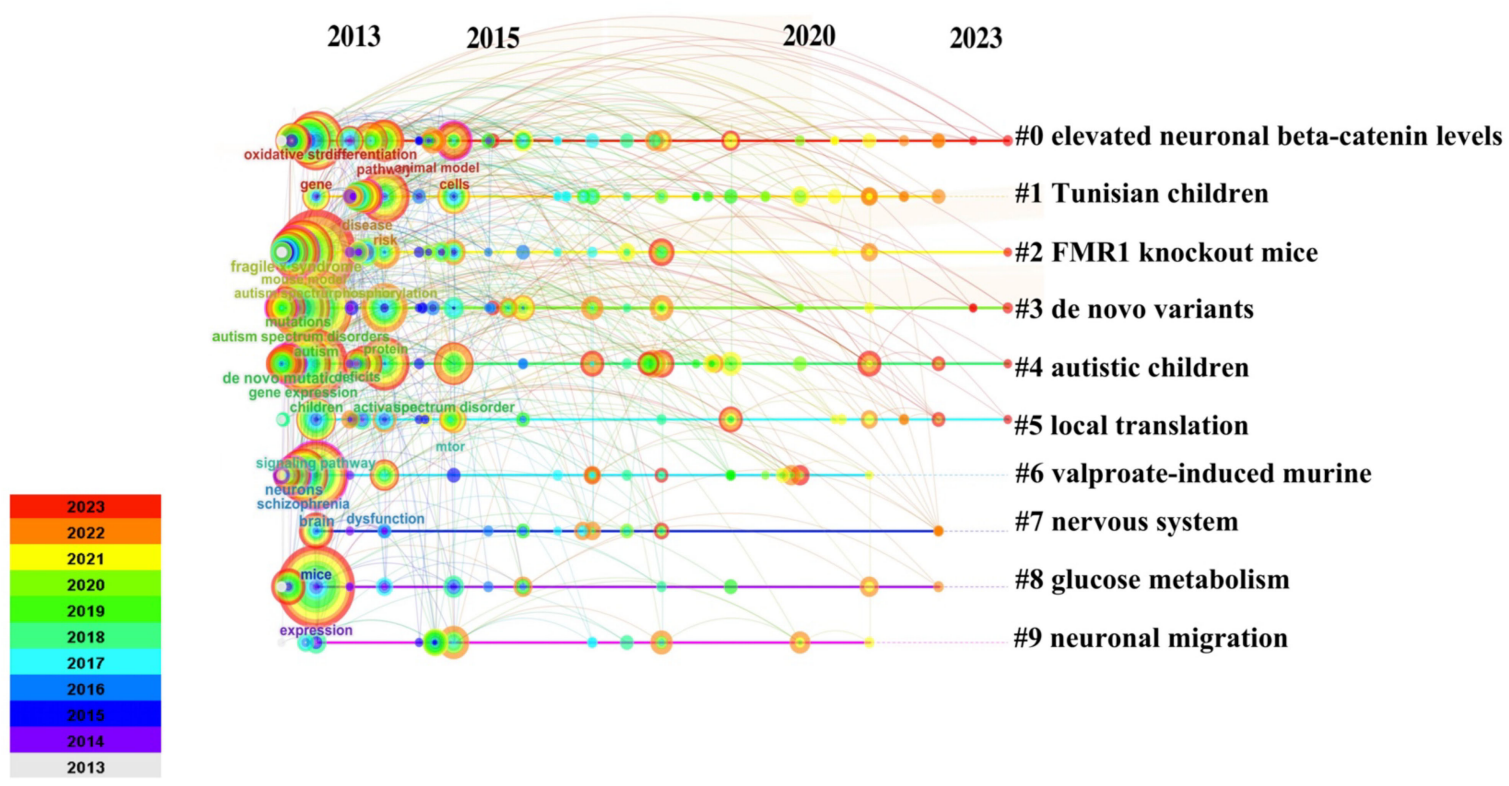
Keyword Clustering Time Graph.

The keyword co-occurrence graph generated by CiteSpace 6.2.R4, as shown in the **Figure 8**, comprises 250 nodes and 739 links. This research involves eight primary keywords, including “elevated neuronal beta-catenin levels,” “Tunisian children,” “ *Fmr1* knockout (KO) mice,” “*de novo* variants,” “autistic children,” “local translation,” “valproate-induced murine,” “nervous system,” “glucose metabolism,” and “neuronal migration.” The clustering of these keywords is evident. The keyword cluster timeline (**Figure 9**) shows that research on ASD-related Signaling Pathways has significantly increased since 2013.

The **Table 6** summarizes the co-occurrence and centrality of the top 5 keywords. “Autism Spectrum Disorder” (170) is the most frequently occurring keyword, followed by “expression” (158), “autism” (3), “children” (117), and “brain” (109). Keywords with significant centrality serve as indicators of critical domains and nodes within the network. The centrality values range from 0 to 1. The top five keywords in terms of centrality are “autism,” “fragile X syndrome,” “mice,” “children,” and “activation.”

**Table 6:** Top five keywords for co-occurrence and centrality.

Ranking	Documents	Keywords	Ranking	Centrality	Keywords
1	170	autism spectrum disorder	1	0.09	autism
2	158	expression	2	0.09	fragile x syndrome
3	150	autism	3	0.09	mice
4	117	children	4	0.08	children
5	109	brain	5	0.08	activation

**Figure 10:**
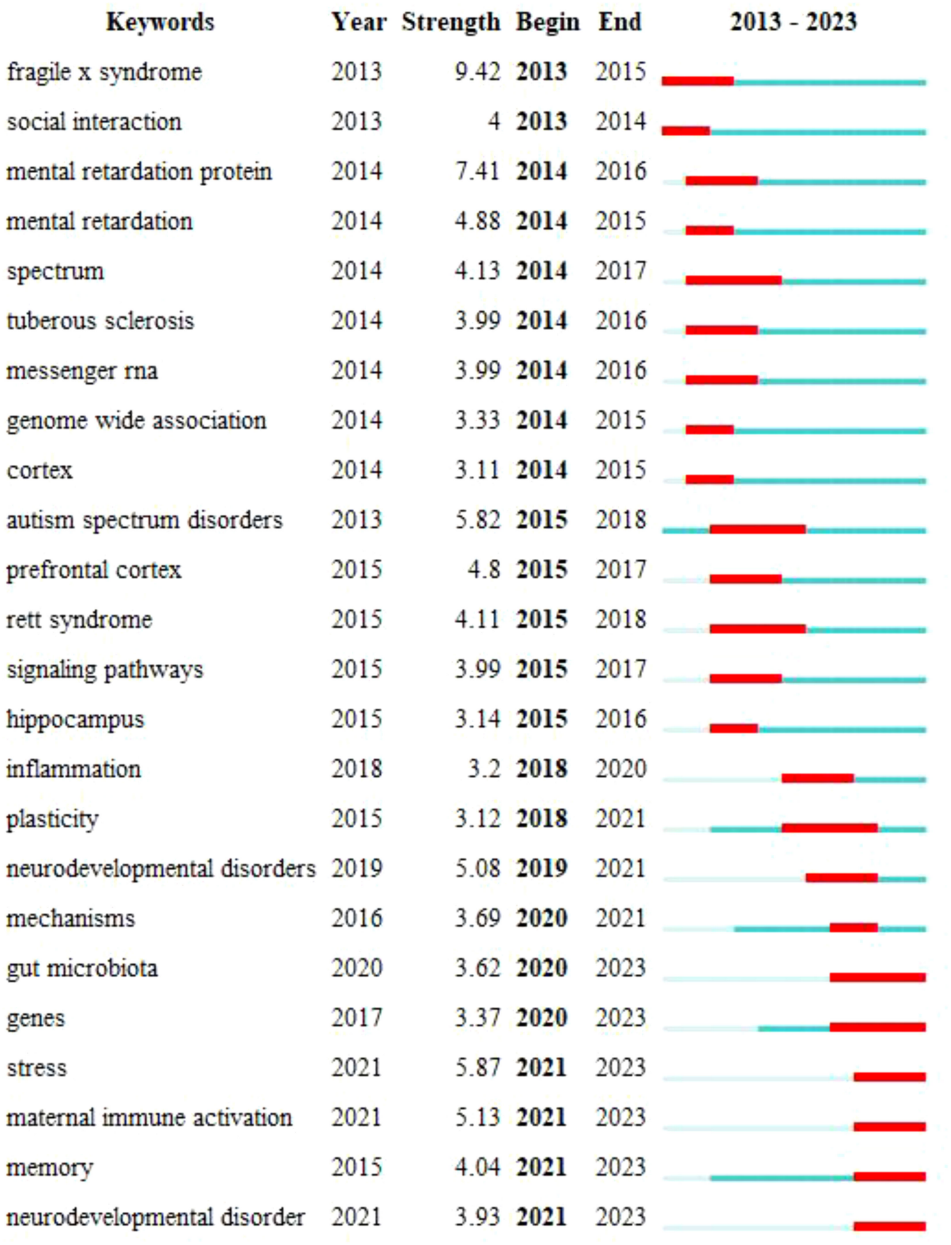
Strongest-citation-bursts. The blue line depicted in the figure signifies the time interval, while the red line represents the period characterized by a surge in keywords.

To gain a deeper insight into the evolving research focus in this field over different periods, an investigation into the evolutionary trajectory through timelines was conducted to assess the appearance of keywords. Using CiteSpace 6.2.R4, a keyword map displaying citation bursts was constructed, enabling the identification of the years in which keyword research started to surge. Ultimately, the top 24 most cited keywords are presented visually (**Figure 10**). Notably, the most recent six keyword citation bursts occurred in 2020 and have continued through 2023.

### 3.7 Associated gene analysis

**Figure 11:**
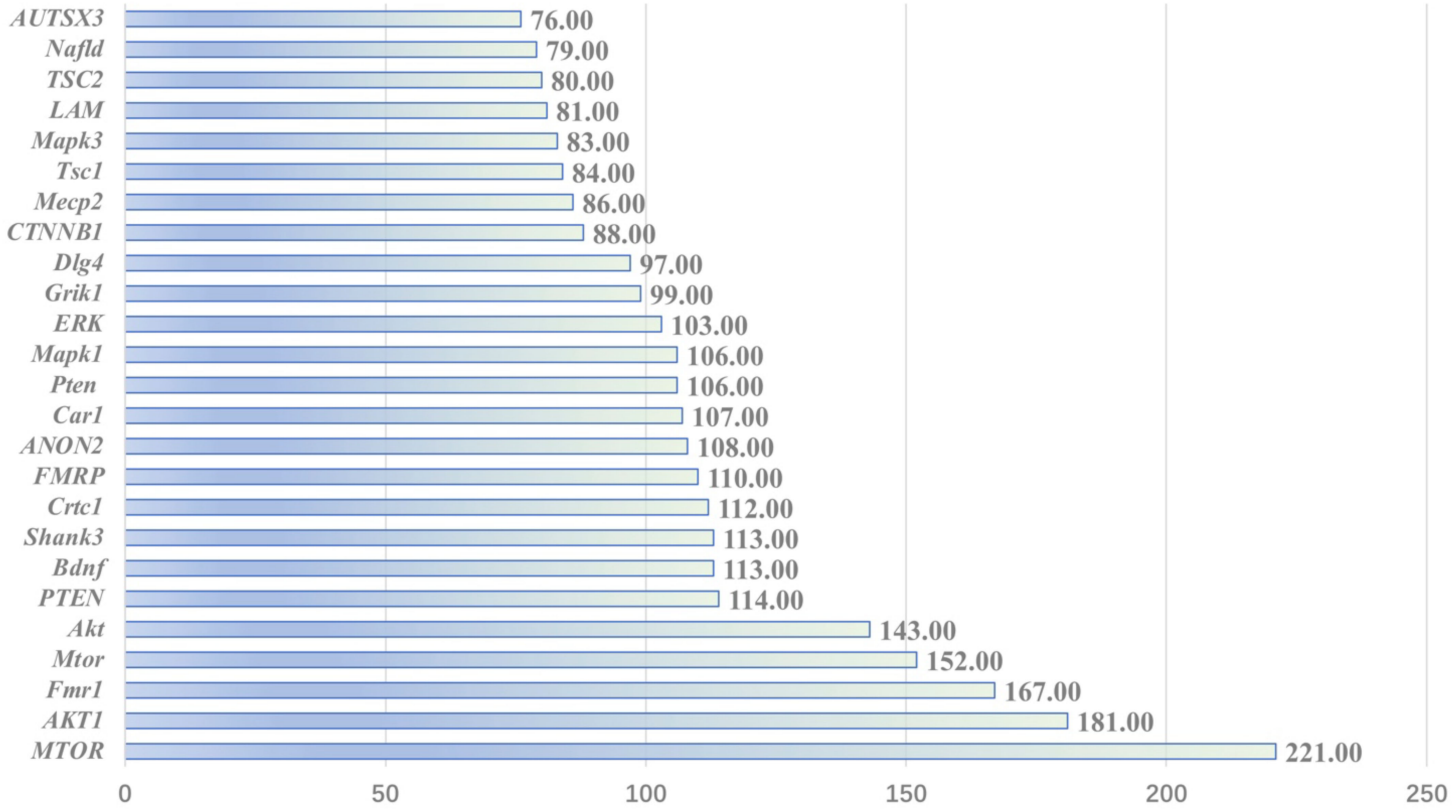
Associated gene analysis.

The BioBERT (29) biomedical language representation model is used to mine and statistically analyze the entity words of genes in the abstract of the article. As shown in the **Figure 11**, *MTOR* has the largest number of documents (221 articles); *AKT1* ranks second, with 181 documents.

## 4 Discussion

While articles on ASD-related Signaling Pathways have garnered widespread attention in recent years, there remain unresolved gaps in understanding the specific pathogenesis of ASD. We analyzed articles related to ASD Signaling Pathways published in the WoSCC database from January 2013 to October 1, 2023. The visual presentation of the knowledge map provides insights into collaborative efforts among countries and institutions, influential authors, cited journals, and research focus areas, thereby revealing the frontiers of this field.

From January 2013 to October 1, 2023, there was an overall increasing trend in annual research publications, especially after 2020, when the literature in this field experienced a sharp increase. This could be attributed to a growing awareness of the importance of ASD Signaling Pathways research, leading to increased funding for research in this domain. For instance, the National Institutes of Health (NIH) in the United States has supported multiple research projects through the Autism Centers of Excellence (ACE) program (30), encompassing various aspects of ASD Signaling Pathways research, including genetics, neurobiology, early intervention, social interaction, and education. The Simons Foundation Autism Research Initiative (SFARI) collaborates with the NIH to support ASD research (31), providing funding that promotes developments in genetics, biology, clinical research, and social sciences related to ASD.

Regarding geographical distribution, most articles originate from the United States, China, Germany, Canada, and the United Kingdom, highlighting their active participation and close collaboration in research, particularly among Western countries (**Figure 3**). It is noteworthy that the United States (intermediary centrality: 0.5), Canada (intermediary centrality: 0.11), and Germany (intermediary centrality: 0.09) have shown exceptional performance in this field. This underscores the leading positions of Europe and North America in ASD-related Signaling Pathways. This phenomenon primarily stems from these countries’ robust capabilities in scientific research, funding support, and technological innovation.

The United States, as a global frontrunner in scientific research, boasts world-renowned research institutions and facilities. For example, the National Institutes of Health’s (NIH) significant investment in ASD research and sustained funding through programs like the Autism Centers of Excellence (32)has propelled the country to a leading position in ASD studies. China, the world’s second-largest economy, has significantly intensified its efforts toward technological innovation in recent years. It has allocated substantial funding and resources to neuroscience, artificial intelligence, and ASD-related research, such as establishing the Institute of Neuroscience and Intelligence Technology and sponsoring numerous projects, significantly contributing to China’s pivotal role in this domain (33). Germany, supported by research grants and government backing, is committed to both basic and clinical ASD research. The German Research Foundation (DFG) (34) and the Federal Ministry of Education and Research (BMBF) (35) have provided crucial financial support, driving Germany’s advancements in this field. The leadership positions of these nations in the ASD-related Signaling Pathways domain underscore their critical role in advancing scientific research and fostering knowledge innovation in this field. Their research outcomes and significant discoveries play a vital role in understanding ASD’s neurobiology and genetics, among other facets, furthering ASD diagnosis, treatment, and prevention research. This leadership also emphasizes the significance of global research collaboration, establishing a firm foundation for propelling global ASD research. However, while these countries dominate this field, it’s essential to acknowledge that achieving global research equilibrium and collaboration still presents challenges. The limited participation of African nations in ASD research poses hurdles to the worldwide understanding and solutions for ASD.

Recent studies indicate that the age-standardized prevalence of ASD in Africa is 1% (95% CI: 0.3-3.1), surpassing the global average of 0.6% (95% CI: 0.4-1%) (36). Access to diagnosis and treatment remains limited due to challenges such as a lack of specialized healthcare professionals and resources, a lack of awareness and understanding of ASD among healthcare providers, and cultural stigma surrounding mental health and developmental disabilities. However, there have been positive developments in recent years. For example, the establishment of the Autism Research Center in Cape Town (37), South Africa, contributes to research initiatives in Africa and beyond to increase awareness and understanding of ASD and promote the rights of people with ASD and their families and interests. The Autism Alliance of Kenya partners with African universities and hospitals to provide training and education in diagnosing and managing ASD (38). The Kenya Autism Alliance also provides online training programs and workshops for African mental health professionals. Funded by NIH, Pauline Samia et al. (39) conducted the first retrospective medical chart review in Kenya. Found that only 44% and 34% of children diagnosed with ASD received speech therapy and occupational therapy, respectively. Notably, seven of the top five institutions, in terms of article quantity, are situated in the USA, making the USA a substantial contributor to this field.

Analyzing widely published works and highly cited journals helps assess the current status of research related to ASD Signaling Pathways. The top ten journals with the highest publication frequencies are concentrated in Europe and the USA. While China plays a substantial role in this field, the absence of well-known Asian publishers underscores the need to cultivate influential international journals. The accepted articles primarily delve into the latest research developments in ASD Signaling Pathways, particularly in brain structure, genetics, and bioinformatics. Molecular Autism stands at the pinnacle regarding publication quantity and citations, establishing its core position in the academic domain.

The author’s collaborative network diagram provides a profound understanding of key figures within this field. Among them, Klann, Eric emerges as the most prolific scholar. His research journey began with a focus on specific issues within Fragile X syndrome, such as dysregulation in translation control and its associations with memory and cognitive function impairments. Gradually, this focus expanded to encompass broader realms of neurodevelopmental and neuropsychiatric disorders. More recently, his studies have delved deeper into the molecular mechanisms of neurological diseases, including gene mutations linked to ASD, investigating their impact on translational function and protein structure (40). He has pioneered optogenetic tools, including cell-specific translation suppression (41), while also exploring gene therapy approaches, particularly targeting gene repeat expansions within Fragile X syndrome (42). His latest publication reveals that mutations in *EEF1A2* result in translational dysfunction and alterations in actin binding, which are associated with autism spectrum disorders, epilepsy, and intellectual disabilities (43).

Delving into frequently cited literature aids in swiftly comprehending pivotal aspects of this field. Notably, the study conducted by Iossifov et al. (26) holds preeminence in terms of co-citation frequency. Through whole-exome sequencing, they unveiled a close association between ASD pathogenesis, *de novo* mutations, and gene copy number variations, with potential variations in the impact of these genetic changes based on affected gender and IQ levels. Furthermore, Silvia De Rubeis et al. (27) work predominantly delves into the genetic architecture of ASD, especially the interplay between common and rare variations and their effects on hundreds of genes, through whole-exome sequencing. Equally significant, Satterstrom FK et al. (28) report the largest-scale ASD whole-exome sequencing study to date, leveraging an enhanced analytical framework that integrates *de novo* mutations and case-control rare variants, leading to the identification of 102 risk genes with a false discovery rate of less than or equal to 0.1. This contribution ranks as the third most frequently co-cited study.

High-frequency keywords are typically employed to ascertain pivotal nodes and pioneering domains within the realm of research. By amalgamating high-frequency keywords, we have generated clusters and constructed a timeline graph, streamlining the analysis of keyword occurrences. Our investigation has revealed that research pertaining to Signaling Pathways in ASD primarily converges on critical terms such as “elevated neuronal β-catenin levels,” “Tunisian children,” “ *Fmr1* knockout (KO) mice,” “novel variants,” “autistic children,” “local translation,” “propionic acid-induced mice,” “neuro system,” “glucose metabolism,” and “neuronal migration.” Analyzing and summarizing these key terms, we arrive at the following conclusions:

### 4.1 Molecular pathways and synaptic plasticity

β-catenin is associated with neuronal connectivity and synaptic plasticity. Studies suggest an elevated level of β-catenin in neurons within the brain tissue of ASD patients (44), which may lead to abnormal activation of the Wnt/β-catenin signaling pathway, subsequently affecting neuronal development and connections (45). This may have implications for functions related to ASD, such as social interaction, communication, and behavior.

Local translation, crucial for neuronal function, involves intraneuronal protein synthesis. *mTOR*, a key regulator, influences various biological processes, including local translation. Its abnormal activation may disrupt local translation, impacting synaptic plasticity and neuronal connectivity, potentially contributing to ASD (46). mTOR also modulates presynaptic activity through different signaling pathways, including mTORC1 and mTORC2, involving various proteins like neurotransmitter receptors and cytoskeletal proteins (47). FMRP, or Fragile X Mental Retardation Protein, regulates neurotransmitter release in CA3 pyramidal neurons by influencing action potential duration (48). Loss of FMRP is associated with defects in presynaptic neurotransmitter release, affecting various neurotransmitters (49). FMRP interacts with specific proteins, such as Staufen, influencing the process of presynaptic neurotransmitter release (50).

### 4.2 Genetics and mutations

The *Fmr1* knockout (KO) mice model has been widely utilized to study synaptic plasticity in ASD, a crucial mechanism within the nervous system linked to functions such as learning, memory, and social interaction. Investigating *Fmr1* knockout (KO) mice also helps unveil molecular pathway abnormalities associated with the absence of *Fmrp*. For instance, *Fmrp* typically influences synaptic function through the modulation of the *mGluR5* signaling pathway (51), and utilizing *Fmr1* knockout (KO) mice can demonstrate abnormal activation of this pathway.

*De Novo* Variants refer to newly occurring gene mutations associated with ASD. These novel variants may encompass gene mutations, insertions, deletions, and other alterations, significantly impacting ASD pathogenesis. Research indicates an association between *de novo* variants in the *CHD8* (chromodomain helicase DNA binding protein 8) gene and ASD pathogenesis (52). The protein encoded by *Chd8* is involved in chromatin remodeling and gene expression regulation (53). Hence, these *de novo* variants may lead to changes in CHD8 protein function, subsequently affecting neuronal development and synaptic function, thereby linking to ASD pathogenesis.

### 4.3 Neuronal development and migration

Neuronal migration refers to the process by which neurons migrate from their point of origin to their final functional location during brain development. This process is critical for the formation of typical neural circuits and brain structure. Aberrant neuronal migration may be linked to ASD pathogenesis. Research has identified the crucial role of *Reelin* protein in neuronal migration, aiding neurons in migrating along the correct pathways to their target regions in the brain (54). Abnormal function of the Reelin gene or the absence of Reelin protein may lead to disturbances in neuronal migration, impacting brain structure and connectivity and thus contributing to ASD pathogenesis.

### 4.4 Drug exposure and metabolism

Researchers have explored its effects on fetal neural development by injecting propionic acid into pregnant mice to simulate a potential environmental factor, i.e., drug exposure, during pregnancy (55). Propionic acid is an antiepileptic drug that has been found to be associated with ASD risk. Investigating the impact of propionic acid on molecular pathways and gene expression (56) helps elucidate the molecular mechanisms related to propionic acid exposure and whether these mechanisms are connected to the pathogenesis of ASD.

Glucose Metabolism is related to brain function and neuronal development, and studying glucose metabolism can reveal metabolic characteristics of ASD. Some studies suggest the presence of mitochondrial dysfunction in ASD patients, and abnormal mitochondrial function may lead to glucose metabolism disturbances (57), thereby affecting neuronal energy supply and normal function. These changes may be associated with some symptoms and characteristics of ASD.

### 4.5 Characteristics of ASD patients

Examining the characteristics and biological foundations of individuals with ASD is essential for a comprehensive understanding of ASD. The term “Tunisian children” refers to a cohort of ASD patients in the Tunisian region (58). These studies concentrate on various aspects of ASD in Tunisia, thoroughly investigating and analyzing multiple dimensions, including biology, cognition, psychosocial factors, and immunology. The research explores the impact of gut microbiota (59) and genomic variations (60) on patients, elucidating challenges in cognitive abilities (61), emotional recognition (62), and familial issues (63). Furthermore, investigations into immunological characteristics contribute to comprehending the pathological mechanisms of autism (64). These profound insights offer crucial indications for a more comprehensive and targeted approach to supporting and intervening in autism.

### 4.6 Nervous system

The nervous system encompasses a broad spectrum of ASD research, including the brain, spinal cord, and neuronal networks, serving as the core system regulating various physiological and behavioral functions. ASD predominantly affects the development and function of the nervous system, including neuronal connections, synaptic plasticity, and signal transmission, among others.

In summary, the aforementioned keywords encompass various aspects of research pertaining to ASD signaling pathways. In comparison to earlier studies, we have observed a shift in the research focus on ASD. Early investigations primarily concentrated on the genetic factors, diagnosis, and behavioral therapies related to ASD, while current research is more directed toward the biological underpinnings of ASD. Recent trends in research also highlight the profound impact of technological advancements on ASD studies. Advancements in techniques such as single-cell RNA sequencing (65), brain imaging technologies [such as fMRI (66) and MEG (67)], and genomics, among others, have provided us with opportunities for a deeper understanding of the mechanisms and characteristics of ASD.

Keyword bursts serve as vital tools for tracking the evolution of academic research hotspots. The research landscape of ASD signaling pathways has undergone rapid evolution from 2013 to 2021, encompassing multiple key themes. The initial focus was centered on genetic disorders associated with autism, such as Fragile X Syndrome and Rett Syndrome. Over time, investigations gradually expanded into broader categories, including social interaction, cognitive functions, and the diversity and complexity within the autism spectrum. Neurological structures, specifically the cerebral cortex and hippocampus, have provided profound insights into the neural foundations of ASD. Additionally, studies on messenger RNA, genomic associations, and signaling pathways have revealed crucial mechanisms related to gene expression, protein synthesis, and intercellular communication. The expanded directions of research involve deeper explorations into neurodevelopmental disorders and mechanisms, along with a growing emphasis on intestinal mechanisms and environmental factors such as maternal immune activation and stress. Overall, this evolutionary process highlights the gradual deepening and broadening of the ASD research field in multiple directions, spanning genetics, neurology, molecular biology, and environmental factors. The entire evolutionary process gradually deepens from surface to depth, progressing from a focus on single genes to the interaction of multiple factors. Over the past two years, keyword bursts have primarily centered around “gut microbiota,” “genes,” “stress,” “maternal immune activation,” “memory,” “neurodevelopmental disorders,” and “stress perception.” Although these are seven distinct high-frequency keywords in our analysis, we have observed their frequent co-occurrence in actual publications. This suggests close associations among these keywords, potentially representing significant relationships and hotspot areas in research.

### 4.7 Gut microbiota

Gut microbiota has emerged as a hotspot in ASD research. It is believed that gut microbiota can influence the neuroimmune system, neural transmission, and brain function (68, 69). Aberrations in gut microbiota may lead to intestinal inflammation and increased gut permeability, which could impact the symptoms of ASD through the gut-brain axis (70).

### 4.8 Genes

Genetic factors play a pivotal role in ASD pathogenesis. ASD is often associated with mutations or variations in multiple genes, and researchers are diligently working to identify these genes and understand how they affect ASD Signaling Pathways and neurodevelopment (71, 72). Genetic research contributes to a deeper comprehension of the genetic basis of ASD.

### 4.9 Stress

Studies suggest that environmental factors may be crucial in ASD pathogenesis. Stress is a common environmental factor that can influence the function of the nervous system and the activity of the immune system, potentially increasing the risk of ASD in specific individuals. Some studies have found abnormalities in the immune system, including enhanced inflammatory responses, in ASD patients (73). Therefore, stress may be linked to ASD Signaling Pathways through immune activation pathways. Additionally, stress can impact neurodevelopment, especially during childhood. Research indicates that childhood stress experiences may adversely affect brain development (74), potentially involving abnormalities in ASD Signaling Pathways.

### 4.10 Maternal immune activation

Researchers investigate how maternal immune activation affects fetal brain development and whether this impact is related to ASD (75). Specifically, this context has piqued interest in inflammation-related cytokines and immune molecules (76, 77). Recent research suggests that the relationship between maternal immune activation and ASD may involve interactions between genetics and the environment. Some individuals may be more susceptible to the effects of maternal immune activation, depending on their genetic background and gene variations.

### 4.11 Memory

Memory is a vital component of neural function and holds significance in studying ASD pathogenesis. Some studies have already discovered abnormalities in neural pathways related to memory in the brains of ASD patients (78). Memory formation and learning processes involve synaptic connections and synaptic plasticity among neurons. ASD Signaling Pathways research also explores factors related to synaptic connections and plasticity (79), as these factors are closely linked to memory function.

### 4.12 Neurodevelopmental disorders

Neurodevelopmental disorders constitute one of the core features of ASD. The Signaling Pathways research encompasses explorations across diverse domains, including imaging assessments, genetic analyses, and comorbidity studies. Neuroimaging has been employed to investigate the associations between brain structure, white matter pathways, functional connections, and neurodevelopmental disorders such as varied white matter changes and functional connectivity patterns observed in ASD, Attention-Deficit/Hyperactivity Disorder (ADHD), and other conditions (80). Furthermore, these studies focus on the correlation between neurodevelopmental disorders and specific genes, copy number variations, such as *KMT2A* variations (81), *SLC6A1*-related (82) neurodevelopmental disorders, and their associations with ASD and ADHD, among others. Simultaneously, there’s a systematic investigation into the comorbidity among different diseases, such as the associations among cerebral palsy, ASD (83), and ADHD. Researchers are dedicated to comprehending the aberrations in neurodevelopmental Signaling Pathways within ASD, aiming to identify potential therapeutic targets and intervention strategies.

In contrast to the past, there has been a gradual shift in focus towards aspects such as gut microbiota, stress, and maternal immune activation. This reflects researchers’ deeper exploration of the interconnections between environmental factors, immunity, and the nervous system. From molecular pathways to neurodevelopment, from genetics to drug exposure, these key terms provide a comprehensive perspective, emphasizing the significance of in-depth exploration in various domains and interdisciplinary research in ASD studies.

This study provides an overview of research related to ASD-associated Signaling Pathways through bibliometric analysis of key indicators such as countries, institutions, journals, authors, and keywords. Researchers have placed particular emphasis on several key areas. Firstly, research focuses on the molecular mechanisms of ASD, including the elevation of β-catenin levels in neurons, local Translation, and glucose metabolism. These processes at the molecular level may be related to the pathogenesis of ASD. Secondly, animal model studies, particularly in *Fmr1* knockout (KO) mice, are used to delve deeper into the biological basis of ASD to identify potential therapeutic avenues. Additionally, population studies occupy a significant position, delving into the diversity of ASD by studying epidemiological characteristics, genetic variations, and behavioral traits in different populations. Lastly, neurosystem research examines the structure and function of the nervous system, including processes like neuronal migration, to unveil the connection between ASD and the nervous system.

Future research directions have been clearly outlined. Firstly, there is a continuous and in-depth investigation into ASD-related genes and neurodevelopmental disorders aiming to unveil their genetic foundations, thereby supporting precision therapies. Simultaneously, there is increasing consensus on the correlation between gastrointestinal microbiota and ASD. However, current evidence solely establishes an association between gut microbiota and ASD due to the observational nature of existing studies and potential confounding variables, lacking causality. Furthermore, the impact of this correlation on ASD prevalence and the specific signaling pathway mechanisms remains unclear, as well as whether this correlation might be disrupted by reverse causality. Therefore, the attention towards gut microbiota is poised to aid in exploring its connection to ASD, potentially offering clues for novel therapeutic approaches. Moreover, future research will delve into understanding individual differences in stress responses among ASD patients, tracking the long-term effects of stress on ASD development to better comprehend the emotional and behavioral traits of ASD patients under stressful conditions. it aims to explore how interventions can improve stress responses in ASD patients. Despite several epidemiological studies linking maternal inflammatory status to neurodevelopmental disorders at an individual level, collective scrutiny has yet to be performed. Future investigations will concentrate on exploring the extensive maternal inflammatory status, its association with various neurodevelopmental disorders in offspring, and its potential mechanisms. Lastly, a deeper understanding of cognitive functions in ASD patients, especially memory and learning functions, will aid in devising personalized therapeutic strategies to enhance the quality of life for these patients. These directions will synergistically contribute to comprehensively understanding ASD signaling pathway research, offering new insights and opportunities for future interventions and treatments.

The analysis provides a comprehensive perspective, outlining the critical Signaling Pathways research within the ASD field. Such comprehensive analysis aids researchers in selecting more forward-thinking and innovative research directions. It helps them comprehend the existing knowledge structure, guiding more targeted studies in areas like gut microbiota, stress, and maternal immune activation. Simultaneously, this study assists policymakers in better understanding the needs and challenges within this field, facilitating the formulation of public health policies and resource allocation. Moreover, this research contributes to fostering collaboration and communication between academia and practitioners (such as physicians, clinical psychologists, etc.). It offers an overview of the latest research findings, enabling them better to comprehend recent advancements and treatment approaches in ASD. This, in turn, improves clinical practices and patient care. Additionally, it provides a common language and platform for researchers and clinical practitioners from different fields, facilitating interdisciplinary collaboration and knowledge exchange within the Signaling Pathways domain of ASD.

## Data availability statement

The original contributions presented in the study are included in the article/supplementary material. Further inquiries can be directed to the corresponding authors.

## Author contributions

KL: Conceptualization, Data curation, Investigation, Methodology, Resources, Software, Visualization, Writing – original draft. JL: Writing – review & editing, Funding acquisition. MC: Supervision, Validation, Writing – review & editing. WL: Supervision, Validation, Writing – review & editing. WZ: Supervision, Validation, Visualization, Writing – review & editing. MH: Formal analysis, Supervision, Visualization, Writing – review & editing. YZ: Supervision, Writing – review & editing. XF: Supervision, Writing – review & editing.
